# Supplementary material for: Flipping the switch on some of the slowest mutating genomes: Direct measurements of plant mitochondrial and plastid mutation rates in msh1 mutants
Source: bioRxiv. 2025 Jan 11:2025.01.08.631957. Preprint. [Version 1] doi: 10.1101/2025.01.08.631957 (PMC11741330; doi:10.1101/2025.01.08.631957)
Supplement: Supplement 4 [file NIHPP2025.01.08.631957v1-supplement-4.pdf]

bioRxiv preprint doi: https://doi.org/10.1101/2025.01.08.631957; this version posted January 12, 2025. The copyright holder for this preprint (which was not certified by peer review) is the author/funder, who has granted bioRxiv a license to display the preprint in perpetuity. It is made available under aCC-BY 4.0 International license.

Table S1. Primers used to validate a sample of mitochondrial and plastid SNVs by PCR amplification and Sanger sequencing. All tested sequences were confirmed as germline variants.

Organelle	MA Line	Position	SNV	Location	F primer (5'→3')	R primer (5'→3')	Marker[1]
Mito	M1_6_F8	21077	C>T	Intron (Rps3)	CTGTTAGCGTAGCTATGCAGTGG	ACTGGTACTTGATTGGGGCG	271
	M3_2_F8	95003	T>C	CDS (Nad2)	CCTGCGTATGAGAACATAGTAATGG	CTTTGACTCTATATATGTGGGCACC	273
	M2_2_F8	191380	C>T	CDS (CcmFC)	CTCGCTTCTTCACAAGCTTACC	TATAGCCACTTCAGCCGTGC	274
	M3_8_F8	123820	A>T	intergenic	AAAGCAAGAAACCCATCCAGC	CAAGTCCTTTTAGGCGGTCC	276
	M3_5_F8	47247	A>G	intergenic	CGCTACACCGCCCTATTGAT	TGTTAGCAGGATCGGGTAGG	278
Plastid	M3_4_F8	67994	A>G	CDS (Rps18)	CAAATTGAGTGCTTGTATGTCAAC	CGAGCTTGTTTTATAGCAATAGTGA	261
	M2_8_F8	61752	T>C	CDS (PetA)	TACCTTATACATCATAACCAAATCAAAGTCG	CTCCGGTAAAATAAGAACAGCTCC	263
	M2_6_F8	73081	T>C	CDS (PsbB)	GCATTTCATGTAACAGGATTATATGGTCC	TCAGCCAGTCCAGCACTAAC	265
	M2_8_F8	6417	C>T	intergenic	CAAGTCGTACGTTGCTTTCTACC	TTCCGTCCCAGAGTACAGTC	267
	M2_5_F8	71585	A>G	Intron (ClpP)	GATTTGCACATATAGGACAAATGAACC	TCTCCCTCGATCGAGATATCC	269

[1]Marker refers to the trace number in the ab1 files for mutant, wild type, and F9 mutant progeny plants (Sanger data available via GitHub: https://github.com/dbsloan/msh1_MA_lines).

bioRxiv preprint doi: https://doi.org/10.1101/2025.01.08.631957; this version posted January 12, 2025. The copyright holder for this preprint (which was not certified by peer review) is the author/funder, who has granted bioRxiv a license to display the preprint in perpetuity. It is made available under aCC-BY 4.0 International license.

Dataset S1. Summary of each organelle SNV.

Dataset S2. Summary of each organelle indel.

Dataset S3. Summary of each nuclear SNV.
